# Medication analysis and pharmaceutical care for a child with Kawasaki disease: A case report and review of the literature

**DOI:** 10.1097/MD.0000000000032488

**Published:** 2023-01-06

**Authors:** Yingqiang Lai, Meirou Feng, Jianrong Deng, Benren Tan, Junfeng Ban, Jinkun Zheng

**Affiliations:** a Department of Pharmacy, Yuebei People’s Hospital, Shaoguan, Guangdong, People’s Republic of China; b Department of Pediatric, Yuebei People’s Hospital, Shaoguan, Guangdong, People’s Republic of China; c Guangdong Pharmaceutical University, Guangzhou, People’s Republic of China; d The Innovation Team for Integrating Pharmacy with Entrepreneurship, Guangdong Pharmaceutical University, Guangzhou, People’s Republic of China.

**Keywords:** clinical pharmacy, Kawasaki disease, pediatrics, pharmaceutical care

## Abstract

To explore the ideas and methods of clinical pharmacists regarding drug therapy for children with Kawasaki disease.

**Methods:**

By participating in a whole drug treatment process for a child with Kawasaki disease, the rationality of the drug treatment plan was analyzed, pharmaceutical care was provided for the child, and a pharmaceutical care model suited to this child was developed.

**Results:**

After treatment, the child was discharged from the hospital, and all signs and major inflammatory indicators returned to normal. The child’s parents were instructed to bring medication, visit regularly, and adjust medication.

**Conclusion:**

Through the entire process of pharmaceutical care, clinical pharmacists are able to identify and resolve drug treatment-related issues in a timely manner, and also make suggestions on rational drug use, which can improve the safety and compliance of drug use in children and the quality of clinical drug treatment.

## 1. Introduction

Kawasaki disease (KD) is an acute vasculitis in children that causes coronary aneurysms in about 25% of untreated cases. KD, also known as cutaneous mucosal lymph node syndrome, is difficult to be diagnosed due to the absence of a direct and rapid detection method. The disease is characterized by its rapid onset, great harm, severe complications, poor prognosis, and other clinical features, and its clinical treatment is difficult.^[1,2]^It has been reported globally and is the leading cause of acquired heart disease in children in developed countries.^[3]^ KD typically affects children under 5 years of age, and the incidence ratio of males to females is 1.7:1.^[4,5]^ In East Asia, the prevalence of KD is extremely high, and it is increasing.^[6–8]^ In recent years, Beijing and Shanghai have published data indicating that there are more than 100 new cases of KD per 100,000 children aged 0 to 4 each year.^[9,10]^ An epidemiological study^[10,11]^ in Shanghai showed that the rate of KD in children under 5 years of age increased from 30.3/100,000 in 2008 to 107.3/100,000 in 2016 and KD is now the leading cause of acquired heart disease in children. Currently, it is believed that the pathogenesis of the disease is caused by the abnormal activation of the immune system and the cascade amplification effect of inflammatory factors, which are triggered by the invasion of bacteria into the bodies of susceptible children and the activation of immune cells.^[12,13]^ At the same time, there are relevant guidelines^[3]^ and consensus^[9]^ for the diagnosis and treatment of KD at home and abroad, and 17 recommendations have been accepted for the diagnosis and treatment of KD in children. These recommendations provide the latest evidence-based recommendations for the diagnosis and management of KD. However, treatment decisions should be tailored to the unique circumstances of individual children. Children are still growing, their bodies are not fully developed, and their drug absorption, distribution, metabolism, and excretion systems differ significantly from those of adults^[14]^; therefore, pediatric pharmacy services must pay more attention to children. Rationale of drug use and drug risk factors may be implemented in the process of disease diagnosis and treatment of children’s drugs benefit maximization, thereby enhancing the overall treatment effect for children.

In this paper, the clinical pharmacist monitored, analyzed, and summarized the medication situation of a child with KD during hospitalization in our hospital, aimed to explore a pharmaceutical care practice model suitable for children with KD and provided the basis for evidence-based pharmacy in the treatment of KD.

## 2. Case information

The child, a 3-year-old male weighing 15 kg, was born naturally with no genetic disease history in his family. Since the onset of the disease, the child’s mental stomach intake and urine and stool have been normal. The hospital admitted him on May 19, 2022. Half a month before admission, he had chapped lips without apparent cause, a red maculopapular rash and other rash reactions with mild pruritus, but no fever, vomiting, or diarrhea. The exact diagnosis and treatment he received at the local health center were unknown, but the rash had subsided and chapped lips remained. Three days prior to admission, the child developed a rash with the same appearance as before, as well as acral and perianal desquamation on both upper limbs; however, his family did not administer any special treatment. The child developed fever with a maximum temperature of 38.6°C, no chills or convulsions, occasional cough, cyanosis, and nasal obstruction 1 day prior to admission. The child was brought to the outpatient department of our hospital for further diagnosis and treatment. The outpatient division planned to ask, “Is the cause of fever Kawasaki disease? And admitted to the hospital.

Blood index common leukocyte elevation predominantly neutrophil elevation, relative lymphocyte reduction. White blood cell count increased to 23.43 × 10^9^/L, platelet count increased to 459 × 10^9^/L, hypersensitivity, C-reactive protein increased to 13.58 mg/dL, and erythrocyte sedimentation rate increased to 40.0 mm/h, according to blood indices. The laboratory examination results of the admitted children are presented in Table 1.

**Table 1:** Main laboratory examination indicators of children admission.

Examination indicators	Value of monitoring	Range of reference
White blood cells (×10^9^/L)	23.43↑	4–10
Hemoglobin (g/L)	126.0	120–160
Platelet (×10^9^/L)	459↑	100–300
Granulocyte rate (%)	77.60↑	50–70
Lymphocyte rate (%)	16.50↓	20–40
Erythrocyte sedimentation rate (mm/h)	40.0↑	0–15
C-reactive protein (mg/dL)	13.58↑	0–3
Procalcitonin (ng/mL)	0.996↑	0–0.05
Direct bilirubin (mol/L)	0.80↓	1–6
Lactate dehydrogenase (U/L)	362.00↑	60–240
Hydroxybutyrate dehydrogenase (U/L)	314.00↑	72–182
Creatine kinase isoenzyme (U/L)	63.60↑	1–24

According to the patient’s medical history, physical examination, and auxiliary examination, the following admission diagnosis was determined: First, KD; second, acute bronchitis; and third, myocardial damage.

### 2.1. Diagnosis basis

Kwasaki Disease: the child was diagnosed with KD after being admitted with “chapped lips, recurrent rash, and fever for one day.” Physical examination of children with rashes and chapped lips reveals a large number of red maculopapular rashes with mild itching that can fade with pressure. Both eyes are affected by conjunctival congestion. Right submaxillary lymph node enlargement, approximately 1 × 1 cm, tenderness, moderate quality, low activity, absence of adhesion, chapping, strawberry tongue, perianal skin flushing, desquamation. Desquamation of both upper limbs. C-reactive protein, elevated erythrocyte sedimentation rate; Acute bronchitis: the child had fever, occasional cough, and physical examination revealed throat congestion and bilateral tonsil I enlargement. Breathe freely. Both lungs produce audible breathing sounds. No rales, wet or dry. Lung texture thickening can be diagnosed alongside chest radiography. Myocardial damage: myocardial damage can be determined if a child’s creatine kinase isoenzyme level exceeds 2 times the normal value.

### 2.2. Main course of treatment

On 19 May, the patient was admitted and administered intravenous immunoglobulin (IVIG) 2 g/kg/d, once IV drip; Ceftriaxone sodium 1.2 g QD; Yanhuning 150 mg QD; Inosine Injection 0.1 g QD; Vitamin B6 Injection 50 mg QD. 20 May, when the fever peaked at 39.5°C, aspirin enteric-coated Tablets 200 mg q8h and Sucralfate Oral Suspension 3 mL q8h were administered. On May 21, the child had no fever, the rash had significantly subsided, and a small amount of red maculopapular rashes were dispersed across the body. The conjunctiva of both eyelids were mildly swollen, and the right submaxillary lymph nodes were enlarged by approximately 1 × 1 cm without tenderness. The lips were no longer chapped, the tongue and pharynx were congested, and the bilateral tonsils were significantly enlarged. A small amount of perianal and acral are used to maintain the regimen of medication. On May 23, the child had no fever, the rash had subsided, the conjunctival membranes of both eyelids were clear, the strawberry tongue had improved, the pharynx was hyperemic, and the bilateral tonsils exhibited grade I enlargement. The inflammation index returned to a normal level, whereas the platelet count remained elevated at 427 × 10^9^/L. The aspirin dose was adjusted to 20 mg/kg/d, while the other medications remained unchanged. On May 24, the child had no fever, the rash had subsided, the right submaxillary lymph node was approximately 0.5 × 1 cm in size without tenderness, and a strawberry tongue was observed. He was discharged. Take the following medications upon discharge: aspirin enteric-coated tablets 100 mg q8h, sucralfate oral suspension 3 mL q8h, and cefdinir dispersible tablets 50 mg tid. The child was revisited on June 7. There was no pharyngeal hyperemia, desquamation of the extremities, redness of the lips, lymph node enlargement, or heart murmur. The platelets were well controlled, so aspirin enteric-coated tablets 100 mg BID were prescribed. On June 21, the patient was seen again, and the color Doppler ultrasound revealed no coronary artery dilation; as a result, the dose of aspirin was reduced to 100 mg QD. The patient was seen again on August 23, and the color Doppler ultrasound revealed that the coronary artery had not expanded. Aspirin may be discontinued.

## 3. Drug analysis and pharmaceutical care

IVIG was prescribed as immunotherapy treatment. Aspirin enteric-coated tablets are anti-inflammatory and antiplatelet aggregation. Sucralfate oral suspension safeguards the digestive tract. Both ceftriaxone sodium and yanhuang are anti-infective. Inosine injection safeguard the heart and other organs. Clinical pharmacists provided medication support to clinicians by searching relevant literature, establishing clinical medicine history, combining the characteristics of the children with medication recommendations, and observing the children’s drug response, while the children were undergone the whole medical care. The main medications administered to children during treatment are listed in Table 2.

**Table 2:** Main medication of children during treatment.

Drug	May 19	20^th^	21^st^	22^nd^	23^rd^	24^th^	June 7	June 21	August 23
IVIG	30 g, once								
Aspirin enteric-coated tablets		200 mg, q8h				100 mg, q8h	100 mg, bid	100 mg, qd	
Sucralfate oral suspension		3 mL, Taking before aspirin							
Ceftriaxone sodium	1.2 g, qd								
Yanhuning	150 mg, qd								
Inosine injection	0.1 g, qd								
Vitamin B6 injection	50 mg, qd								
Wuweita oral liquid	1.5 mL, tid								
Cefdinir dispersible tablets						50mg, q8h			

IVIG = intravenous immunoglobulin.

### 3.1. anti-inflammatory, antipyretic, and immune

Treatment plan: IVIG 2 g/kg/d, once IV drip, and aspirin enteric-coated tablets 200 mg q8h.

Reasons: On the second day after admission, the child’s fever peaked at 39.5°C. aspirin’s effects include antipyretic, anti-inflammatory, and antiplatelet aggregation. Because it regulates the activity of immune proteins and immune cells and prevents the spread of inflammatory cells to endothelial cells, IVIG is suitable for treating autoimmune diseases. The most recent consensus^[9]^ in China suggests IVIG combined with aspirin as the primary treatment for KD.

Rationality analysis and pharmaceutical care: Aspirin enteric-coated tablets: compared to aspirin ordinary tablets, its enteric-coated tablets are less irritating to the stomach, and the use of enteric-coated tablets reduces the gastrointestinal reaction of children. The blood admission item revealed that the children’s platelet count rose to 459 × 10^9^/L and the erythrocyte sedimentation rate accelerated to 40.0 mm/h, so aspirin 40 mg/kg/d was administered as shock therapy. The clinical pharmacist monitored the child’s body temperature, platelets, and inflammatory markers and promptly adjusted the child’s aspirin dosage to 20 mg/kg/d, 10 mg/kg/d, and 5 mg/kg/d. The child was reexamined 3 months after being released. All indicators were normal, and the color Doppler ultrasound revealed no abnormalities; as a result, aspirin was discontinued. According to treatment guidelines,^[3,9]^ the clinical pharmacist selected aspirin combined with IVIG for the treatment of KD, which can reduce the rate of coronary artery dilatation, improve blood indicators, and boost the immune system. The drug was immediately discontinued after the child developed chills, cold hands and feet, and other adverse reactions approximately 10 minutes after IVIG infusion. Injections of dexamethasone sodium phosphate were administered for symptomatic treatment. As soon as the child’s body temperature returned to normal, the pharmacist suggested discontinuing intravenous IVIG, and the physician believed the adverse reaction was due to inflammation and fever. The pharmacist concurred with the physician. There were no adverse reactions until the end of the intravenous drip. The value of the clinical pharmacist–physician cooperation system is well reflected by this procedure. When combined with clinical pharmacist’s medication support, clinicians with extensive diagnostic experience can provide superior patient care.

### 3.2. Treatment for respiratory tract infections and pharmaceutical care

Treatment plan: Yanhuning 150 mg QD, Ceftriaxone 1.2 g QD, and Cefdinir Dispersible Tablets 50 mg TID after discharge.

Reasons: The use of antiviral drugs in conjunction with febrile symptoms and inflammatory markers in children, Yanhuning, is favored for the treatment of viral infections and is often used to treat children with pneumonia. The child’s physical examination revealed chapped lips, throat congestion, a strawberry-like tongue, tonsils, and other symptoms. Yanhuning can rapidly alleviate the symptoms of acute upper respiratory tract infection, reduce fever rapidly, shorten the duration of treatment, and have a clear curative effect.^[15]^ Cefdinir dispersible tablets and Ceftriaxone sodium have an antibacterial effect and can be used to treat bacterial infections of the respiratory tract in children with tonsil inflammation, sore throat, and other symptoms.

Rationality analysis and pharmaceutical care: The use of Yanhuang^[16]^ in clinical settings is safe and reliable, with no adverse reactions and reasonable usage and dosage. Ceftriaxone sodium: Taking into account the physical symptoms and laboratory findings of the clinical pharmacist and physician, they decided to administer Ceftriaxone sodium for antimicrobial treatment and application in 2 days with no fever and a controlled inflammation index. In accordance with the instructions to continue medication for 48 to 72 hours after fever, taking the experience to treat acute disease, there was a lack of rationality, but the condition of children with fever improved obviously. Two days later, sputum culture revealed the presence of 2 + Streptococcus pyogenes. A clinical pharmacist monitored the entire process, reviewed fever, cough, and other discomfort reactions, and evaluated drug sensitivity results. Cefdinir dispersible tablets: Taking into account the recurrence of fever, the doctor prescribed streptococcal antibacterial drugs. The drug’s dosage was 50 mg q8d, and the duration of treatment was 3 days, with reasonable usage and dosage. The drug was a time-dependent antibacterial drug with a uniform administration time interval and an enhanced effect. There were no bacterial colonies. The results of the sputum culture revealed normal bacterial growth. Throughout the period, clinical pharmacists monitored the children for fever and adverse reactions and provided medication information to the children’s families.

### 3.3. Analysis of rash treatment medications

Treatment plan: Vitamin B6 Injection 50 mg QD, Wuweita Oral Liquid 1.5 mL TID.

Reasons: Vitamin B6 injections and Wuweita Oral Liquid can be used to treat rashes and chapped lips for the following reasons: Vitamin B6 deficiency can result in seborrheic dermatitis, which affects the skin around the face and mouth and can spread to other parts of the body. Children’s clinical manifestations included chapped lips, glossitis, and oral inflammation, so the clinical pharmacist selected these medications as treatment.

Rationality analysis and pharmaceutical care: Vitamin B6 injection is a high-risk medication that, if administered improperly, can cause severe harm to patients. Additionally, dexamethasone sodium phosphate injection can inhibit vitamin B6 activity.^[17]^ Furthermore, vitamin B6 cannot be used for an extended period of time. The pharmacist advises caution and physician adoption. The clinical pharmacist researched relevant medication guidelines and other information regarding Wuweita Oral Liquid. This drug can be used to treat skin rash caused by vitamin B deficiency and has strong efficacy; it is recommended for use; the physician accepted and adjusted the single dosage to 1.5 mL based on the child’s condition; usage and dosage are not only reasonable, but can also stimulate the child’s appetite.

### 3.4. Analysis of medications used to treat myocardial injury

Treatment plan: Inosine injection 0.1 g QD

Reasons: KD can be complicated by coronary artery disease, which is the leading cause of death in children,^[18,19]^ so heart protection is necessary. Inosine injection is a drug that stimulates the energy metabolism of cells. It is an adjuvant treatment for leukopenia, thrombocytopenia, cardiac insufficiency, and other diseases, and it can boost immunity, so it can be used to treat coronary heart disease.^[20,21]^ Three of the myocardial enzymes, including the creatine kinase isoenzyme, were significantly elevated based on laboratory testing. The specific data is shown in Table 1. Injections of inosine were used to prevent cardiac damage.

Rationality analysis and pharmaceutical care: Inosine injection specification was 0.1 g:2 mL/branch-04 (JB). The physician and the pharmacist adjusted the dosage based on the child’s condition, and 0.1 g of inosine injection was administered intravenously at a single dose.

### 3.5. Analysis of medications used for gastric mucosal protection

Treatment plan: Sucralfate Oral Suspension 3 mL to be administered prior to aspirin.

Reasons: The clinical pharmacist consulted the literature^[22,23]^ and discovered that many children with aspirin-induced gastrointestinal bleeding symptoms. Aspirin inhibited COX-1, decreased the production of mucosal prostaglandin PG, altered the mucin and phospholipid secretion by mucosal cells, and weakened the gastric mucosal barrier. Aspirin can also inhibit platelet COX synthesis, reduce thromboxane A_2_ synthesis, inhibit platelet aggregation, and cause bleeding. Additionally, some researchers believe that gastrointestinal bleeding may be one of the complications of KD.^[24,25]^ Sucralfate oral suspension was therefore administered to protect the gastric mucosa and duodenum.

Rationality analysis and pharmaceutical care: sucralfate oral suspension is a gastric mucosa protective agent that can reduce the irritation of aspirin on the stomach and prevent adverse gastrointestinal reactions such as gastrointestinal bleeding. It is not appropriate for long-term use, as it will cause phosphorus loss in body fluids. Physicians accepted the clinical pharmacist’s recommendation that the drug’s dosage should be adjusted based on the timing of aspirin administration.

## 4. Discussion

There are numerous controversies regarding the usage and dosage of IVIG and aspirin in the acute phase of KD. Currently, there is no clear medication guideline for the treatment of KD in China. The following is a discussion of the administration of IVIG and aspirin by the clinical pharmacist who reviewed voluminous literature and expert consensus.

### 4.1. Discussion of IVIG administration timing

The course of KD, defined as the first day of fever, can be divided into 3 stages^[26]^: early stage (fever duration within 4 d), middle stage (fever lasts 5–9 d), and late stage (fever lasts longer than 9 d) In 2004, the American Heart Association stated that early administration of IVIG for KD did not reduce the risk of coronary artery disease and that nonresponse to IVIG was possible. Early Chinese research^[26]^ suggested that the optimal time to administer KD is in the middle of the disease’s progression. And the 2017 update of the American College of Cardiology indicated^[3]^ that IVIG should be administered as soon as possible to patients diagnosed with KD, and the administration of IVIG after 5 days of fever was no longer emphasized; the 2018 European consensus made the same recommendation.^[27]^ Updated in 2022, the Chinese expert consensus on the treatment of KD states^[9]^ that IVIG therapy should be initiated as soon as the diagnosis of KD is confirmed. Based on the diagnostic experience of the attending physician, this child was diagnosed with KD on the second day of the disease’s progression, and the clinical pharmacist was invited to participate in the consultation and formulation of the drug administration plan. Based on the latest domestic and international medication guidelines^[3,9,27]^ and the humanistic spirit of early diagnosis and early treatment in medical ethics, the pharmacist suggested administering IVIG immediately for immunotherapy.

### 4.2. Discussion of the IVIG administration

The IVIG dosage is 2 g/kg. In China, there are currently 3 primary IVIG administration methods^[9]^ for the treatment of KD: 2g/kg/d, once IV drip, 1 g/kg/d, twice IV drip, and 200 to 400 mg/kg/d, multiple IV drip. Studies^[28,29]^ suggest that the therapeutic effect of KD is positively correlated with the IVIG dose, and the greater the IVIG dose in a single infusion, the greater the therapeutic effect. Yang Haijuan^[30]^ conducted a retrospective analysis on 100 children with KD and found that the single high-dose group had a significantly higher treatment effectiveness rate than the multiple low-dose group. Multiple low-dose uses could easily lead to platelet aggregation, increase blood viscosity, and increase the risk of thrombosis, whereas a single high-dose shock of IVIG could quickly reach the effective concentration of blood drug, allowing for better symptom control. Mi^[31]^ analyzed 178 children with KD statistically and found that different administration methods of IVIG had no significant effect on the recovery of children’s body temperature, and that a single large dose of IVIG caused headache, palpitations, nausea, and other adverse reactions in some children. Therefore, it was suggested to administer 1 g/kg/d via IV drip for 2 consecutive days. Xu Haiyan meta-analysis^[32]^ revealed that different IVIG administration methods had comparable effects on the incidence of coronary artery disease and the recovery of clinical symptoms, signs, and inflammatory markers in children, and that the differences were not statistically significant. According to the 2022 expert consensus,^[9]^ a single dose of 2 g/kg/d should be administered if the intravenous infusion time within 10 hours is used as the control standard. For the intravenous infusion time over 10 hours, the drug was administered at a dosage of 1 g/kg/d via IV drip twice daily for 2 consecutive days.

The pharmacist reviewed a large number of reports on the use of IVIG in the treatment of KD both domestically and internationally, as well as the most recent domestic guidelines, and determined the administration method to be 2 g/kg/d, once via IV drip. The drug was immediately discontinued when the child developed sudden chills, cold hands and feet, cyanosis, and other adverse reactions approximately 10 minutes after intravenous administration. After the child’s adverse reactions subsided, the pharmacist recommended discontinuing this administration and adjusting the dosage to 200 to 400 mg/kg/d via multiple IV infusions. The physician believed that the symptoms, including chills, were due to inflammation and fever. Considering the cost of the medication, the physician recommended continuing IV drip administration and closely monitoring vital signs. The pharmacist concurred with the physician’s assessment. Ultimately, no adverse reactions occurred after the IV drip was continued.

### 4.3. Discussion of aspirin administration

As an antipyretic, analgesic, and antiinflammatory medication, aspirin has a favorable effect on platelet aggregation and can significantly enhance hemodynamics.^[33]^ The dosage of aspirin during the acute phase of KD remains controversial both domestically and internationally. The American Heart Association^[3]^ suggests that high-dose aspirin (80–120 mg/kg/d) should be administered during the acute phase of KD, and then switched to low-dose aspirin (3–5 mg/kg/d) after fever reduction. If the child does not have coronary artery disease, low-dose aspirin should be continued for 6 to 8 weeks following the onset of symptoms. If the child has coronary artery disease, aspirin should be administered until the echocardiogram reveals that the heart is normal. Latest domestic expert consensus^[9]^ recommends that moderate-doses of aspirin for acute treatment of KD, antifebrile review 48 to 72 hours after inflammatory indexes, if back to normal, aspirin reduction to 3~5 mg/kg/d, for no coronary artery lesions or coronary artery for children with mild expansion but recover within 30 days, should be taken for 2 to 3 months. Hu^[34]^ searched the most recent database based on the principles of evidence-based medicine and discovered that there was no difference in the proportion of coronary abnormalities between children who received a moderate dose of aspirin and those who received a high dose of aspirin among KD children who received IVIG and Aspirin combined treatment. This suggests that a moderate or high dose of aspirin during the acute phase of KD has no impact on the prognosis of children.

Based on expert consensus and literature reports, clinical pharmacists in our hospital recommended the selection of 100 mg/tablet, 2 tablets q8h, which corresponds to the moderate dose of aspirin, 40 mg/kg/d. The patient’s inflammatory index returned to normal 48 hours after the antipyretic examination, while the platelet count decreased slightly but remained above the normal range at 427 × 10^9^/L. The physician accepted the clinical pharmacist’s recommendation to reduce the patient’s aspirin dosage to 20 mg/kg/d. The patient was reevaluated 2 weeks after being discharged. The inflammatory markers and platelet count returned to normal, and the color Doppler ultrasound revealed no abnormalities. The physician accepted the pharmacist’s suggestion to reduce the aspirin dosage to 10 mg/kg/d. One month after discharge, the child was reexamined, and all indicators were normal. The physician accepted the pharmacist’s suggestion to reduce the dosage of aspirin to 5 mg/kg/d. Three months after the child’s discharge, all vital signs were normal, and the medication could be discontinued.

### 4.4. Discussion on the superiority of the clinical pharmacist-physician cooperation model

China’s clinical pharmacy is still in its early stages of development.^[35]^ Clinical pharmaceutical care is the “patient-centered” pharmaceutical practice service provided by pharmacists in clinical practice; it exemplifies the value of pharmacists. The mode of pharmacist–physician collaboration focuses on clinician diagnosis and treatment. Clinical pharmacists maximize their professional advantages, provide reasonable drug treatment plans to physicians, provide quality pharmaceutical care services to patients, and assist physicians in resolving drug treatment inconsistencies during the course of treatment in order to optimize drug administration plans and avoid medication risks. Since the patient was admitted between May 19 and May 21, the infection was under control and the fever was under control within 4 days (the fever started the day before the patient was admitted). After receiving the physician-initiated consultation, the clinical pharmacist immediately consulted a large amount of literature and expert consensus on the treatment of KD, formulated the initial medication plan with a focus on the treatment of KD and infection, and administered symptomatic treatments, such as rash, heart protection, and gastrointestinal protection. During the period of pharmaceutical care, the dosage of aspirin should be adjusted based on platelet and related inflammatory indications to reflect rational drug use and to ensure that children receive safe and effective treatment.

## 5. Conclusion

In this paper, a clinical pharmacist provided full pharmaceutical care to a child with KD complicated by bronchial infection, assisted the clinician in identifying and resolving drug treatment problems in a timely manner, and made recommendations for rational drug use, which increased the safety of medication for children and the level of effectiveness of drug therapy, and shifted the clinical pharmacy practice from “checking evidence” to “creating evidence.” It provides the basis for evidence-based pharmacy in the treatment of KD. In addition, it demonstrates the integrated pharmacist-physician collaboration model based on the interdisciplinary team background and perspective of clinical pharmacy and pediatrics.

## Author contributions

Lai provided pharmaceutical care to the child and drafted the first manuscript. Feng made a contribution to acquisition and interpretation of data. Deng was in charge of the child and provide clinical guidance. Ban revised the language and grammar of the manuscript. Zheng and Tan revised the manuscript that led to the final approval of the current submission.

**Drug curation:** Yingqiang Lai.

**Formulation analysis:** Yingqiang Lai, Meirou Feng.

**Methodology:** Junfeng Ban.

**Resources:** Jinkun Zheng.

**Supervision:** Jinkun Zheng, Benren Tan, Jianrong Deng.

**Validation:** Jinkun Zheng, Benren Tan.

**Writing—original draft:** Yingqiang Lai.

**Writing—review and editing:** Junfeng Ban.
